# Full-scale network analysis reveals properties of the FV protein structure organization

**DOI:** 10.1038/s41598-023-36528-z

**Published:** 2023-06-12

**Authors:** André J. Ferreira-Martins, Rodrigo Castaldoni, Brenno M. Alencar, Marcos V. Ferreira, Tatiane Nogueira, Ricardo A. Rios, Tiago J. S. Lopes

**Affiliations:** 1 Center for Advanced Analytics, Itaú Unibanco, São Paulo, Brazil; 2 Institute of Computing, Federal University of Bahia, Salvador, Brazil; 3 Center for Regenerative Medicine, National Centre for Child Health and Development Research Institute, 2-10-1 Okura, Setagaya, Tokyo 157-8535 Japan

**Keywords:** Protein sequence analyses, Machine learning

## Abstract

Blood coagulation is a vital process for humans and other species. Following an injury to a blood vessel, a cascade of molecular signals is transmitted, inhibiting and activating more than a dozen coagulation factors and resulting in the formation of a fibrin clot that ceases the bleeding. In this process, the Coagulation factor V (FV) is a master regulator, coordinating critical steps of this process. Mutations to this factor result in spontaneous bleeding episodes and prolonged hemorrhage after trauma or surgery. Although the role of FV is well characterized, it is unclear how single-point mutations affect its structure. In this study, to understand the effect of mutations, we created a detailed network map of this protein, where each node is a residue, and two residues are connected if they are in close proximity in the three-dimensional structure. Overall, we analyzed 63 point-mutations from patients and identified common patterns underlying FV deficient phenotypes. We used structural and evolutionary patterns as input to machine learning algorithms to anticipate the effects of mutations and anticipated FV-deficiency with fair accuracy. Together, our results demonstrate how clinical features, genetic data and in silico analysis are converging to enhance treatment and diagnosis of coagulation disorders.

## Introduction

Humans and other species are equipped with blood coagulation, a complex and elegant mechanism to stop bleeding following an injury to a blood vessel. In response to injury to the endothelial layer surrounding blood vessels, Tissue factor pathway inhibitor (TFPI) is produced and triggers the sequential activation of more than a dozen other proteins to form a stable fibrin clot^1^. The elements of this highly orchestrated system are vulnerable to perturbations that disrupt its proper functioning. Patients harboring mutations on the coagulation factor 5 gene (F5), develop the so-called FV-deficiency, a rare coagulation disorder causing hemorrhages and uncontrolled bleeding. At present, there are ${\sim }$400 cases described in the medical literature, but given the difficulty of diagnosis and characterization of mutations even in developed countries, this number is likely much higher.

In humans, the F5 gene is located on the chromossome 1 at 1q23, with 80,000 base pairs and 25 exons encoding a 7 kb mRNA^2,3^. The FV protein has 2224 amino acids (2196 in its mature form, after the trimming of a signal peptide at its N-terminus), and 5 domains (A1, A2 A3, B, C1, C2), that with the exception of the B-domain, share $\approx 40\%$ identify with the Coagulation factor VIII (ref.^4^). Apart from a small proportion of FV that is located within the alpha-granules of platelets^5^, it mainly circulates as a single-chain polypeptide of 330 kDa (ref.^5^).

The underlying biology of this protein is fascinating because it exerts opposing functions depending on the stage of its life-cycle. After the coagulation cascade is triggered, thrombin and the activated blood coagulation factor X (FXa) activate FV via a proteolytic cleavage that releases its B-domain. From this point onward, its activated form (FVa) and FXa form the so-called prothrombinase complex, that bind and convert prothrombin to thrombin^6^. The presence of FVa enhances this reaction more than 5-fold^7^, making it a key pro-coagulant factor. Later on, FVa is inactivated by the activated Protein C (APC), that cleaves specific portions of FVa and turns it into FVac, a form that associates with APC and Protein S to inactivate FVIIIa - turning FVac into anti-coagulant molecule^8^.

Patients with FV-deficiency usually do not present a bleeding phenotype as severe as those with hemophilia A or B. Instead, FV-deficiency is associated with bleeding in the oral mucusa, menorrhagia, and hemorrhages after surgical procedures - often where the disorder is diagnosed for the first time^9^. At present, there are no recombinant proteins for prophylactic treatment, instead, patients rely on fresh-frozen plasma products, bypassing agents like recombinant FVIIa, or activated prothrombin concentrates (FEIBA)^10–12^. Although critical for routine therapy and pre- or post-surgery treatment, these alternatives present major drawbacks, like the risk of viral infections, unclear mechanism of action and difficulty in establishing appropriate treatment doses. Hence, it is clear that efficient and affordable therapeutic versions of FV would be beneficial to patients.

In this study, we aimed to understand the fine details of this protein and elaborate a comprehensive map describing the importance of every FVa residue. With this goal in mind, we created a residue interaction network (RIN) of this protein, where the residues are the nodes, and two nodes are connected if they are in close proximity in the protein’s 3D structure. This representation can be explored by network analysis algorithms that are very mature and have been used to identify emerging properties of networks created by humans (e.g., energy grids^13^), as well as biological networks (reviewed in ref.^14^). We identified the critical residues of FV and pinpointed the characteristics of the residues that if mutated, lead to FV-deficiency. Moreover, together with the structural characteristics of this protein, we implemented a machine learning framework (ML) to predict the risk of FV deficiency in patients harboring single-point mutations (we called it the FV-Class). As we verified using the genetic profile of more than 60 patients from different countries, this system predicted with good accuracy the extent which mutations lead to the loss of FV function.

Together, the contributions of our study are two-fold. First, it presents in quantitative terms the importance of the residues of FVa, serving as a roadmap for targeted mutations aiming to improve the activity and stability of FV, and second, the FV-Class framework is the basis of sequence based diagnosis, and demonstrates the feasibility of using artificial intelligence even where the input data is scarce - as is often the case for rare diseases. Importantly, we made the FV-Class code available to the community, anticipating that researchers will repurpose it to study other diseases.

## Results

### The FV protein network

**Figure 1:**
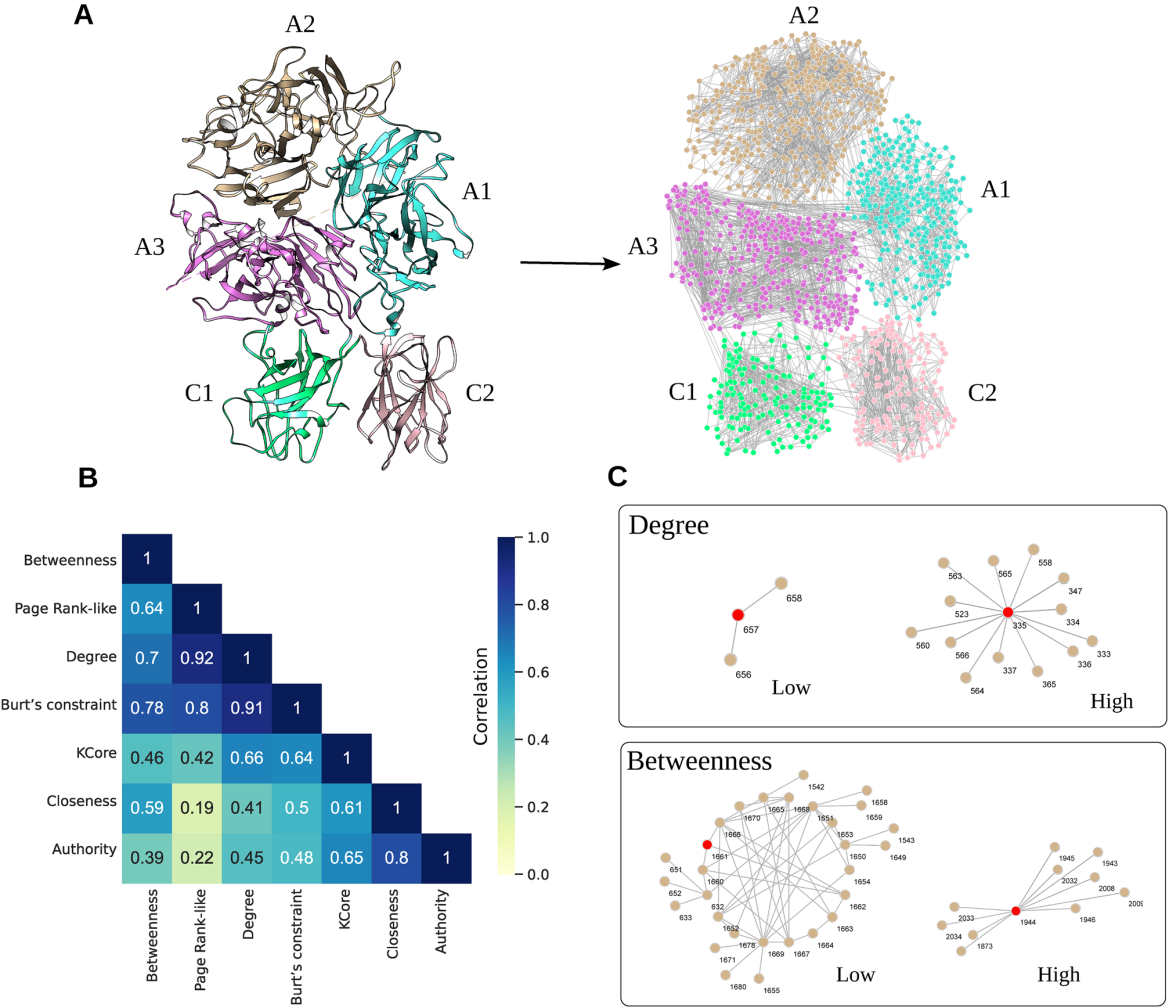
From the protein structure to a residue network. (**A**) The FV protein has 5 domains that share a strong structural similarity to FVIII, albeit they only share ${\sim }$40% of sequence similarity^4^. Using the structure 7KVE as input (ref.^15^), we created a residue network as an undirected unweighted graph, where the residues are the nodes, and two nodes are connected by an edge if they are in close spatial proximity in the three-dimensional space (we named it FV-RIN). (**B**) We calculated multiple measures of centrality to quantify the importance of each node of the FV-RIN. These measures, based on different principles of local and global connectivity, displayed a moderate Spearman correlation to each other, which led us to select only a few well-known measures for the analyses. (**C**) The degree is simply the number of neighbors a node has, and the betweenness counts the number of shortest-paths from every node that pass through a given node. Hence, nodes with high degree and high betweenness values are among the most central residues in the protein structure.

To study the details of the FV protein structure, we create a RIN of this protein using the PDB entry 7KVE as input^15^. We used the Rosetta software package to “relax” the side chains of all its residues (i.e., we searched for the rotamers orientations that minimized the overall free-energy of the structure). Next, we used RINerator (ref.^16^) to create the FV-RIN in three steps. First, hydrogen atoms were added to the structure to allow the identification of non-covalent interactions between residues; these non-covalent interactions were identified using a probe of radius  0.25 Å rolled around the van der Waals surface of each residue. An edge was then established if two non-covalently bonded atoms were touched by the probe; in the last step, these interactions were summarized and composed the edges of the network. Additionally, the interactions between residues were of 3 types, i.e., side-chain - side-chain, side-chain - main-chain or main-chain - main-chain. In the end, the FV-RIN had 1374 nodes, 4416 edges, the distance between interacting residues was ${\sim }5$ Å and all edges were undirected and unweighted (Fig. 1A; Supplementary Table S1 contains the complete FV network and Supplementary Figure S1 contains a comparison to a weighted version of the network).

Previous studies have demonstrated that the centrality measures of residues in a RIN play an important role in the identification of residues that influence protein conformation^17^, interaction with other proteins^18^ and stability^19–21^. Therefore, to identify the key residues of the FV structure, we calculated for each nodes in the network the degree, betweenness, closeness, Burt’s constraint, Page Rank-like, KCore, and Authority Score (see Methods).

Interestingly, we found that although these measures were calculated based on different principles, some of them were correlated to each other in the FV-RIN (Fig. 1B). In practice, this indicates that for further analyses, it suffices to use only simple and well-studied centrality measures. The degree measures the number of neighbors of a given node; the betweenness quantifies how often a node is in the shortest path between all pairs of nodes; and the closeness is the average length of the shortest paths between a given node and all other nodes in the network (Fig. 1C). In terms of protein architecture, the degree is a local measure quantifying the number of atomic interacting partners of a residue, and the betweenness and closeness provide global measures indicating how a given residue contributes to the overall stability and allosteric forces maintaining the structure in place^22–24^. Therefore, these measures can appropriately and concisely describe the FV-RIN.

### Identification of critical residues

**Figure 2:**
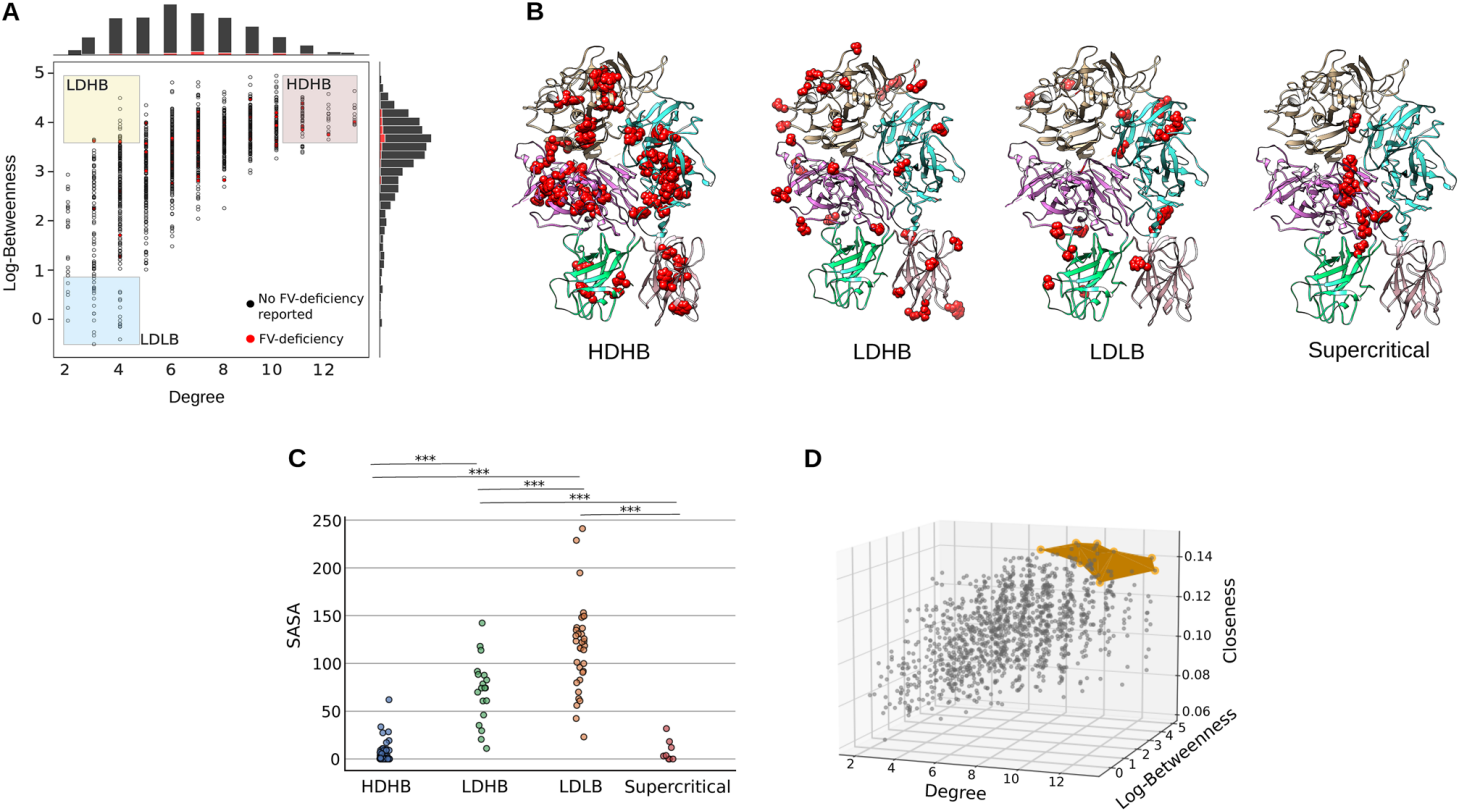
Quantifying the residues’ importance. (**A**) We automatically divided the centrality values into 4 bins to identify the groups highlighted. In the log-betweenness and degree dimensions, the intervals were $(-0.484, 0.886)$ and (1.988, 4.75) for LDLB; (3.613, 4.977) and (1.988, 4.75) for LDHB; (3.613, 4.977) and (10.25, 13.0) for HDHB. (**B–C**) The HDHB nodes are mainly located at the core of all domains of the FV protein structure, and play a pivotal role in maintaining the structure in place via attractive and repulsive atomic forces. The LDHB nodes are those that despite being connected to only a few nodes, are central to the long-range communication network formed by all residues. Finally, the LDLB residues are located at the surface of FV, with only a few connections and are usually not conserved (Supplementary Tables S2–S3). (**D**) The closeness centrality is the inverse of the steps necessary to reach every other node in the FV-RIN. Therefore, the most central residues of the protein structure display high closeness values. Using the Pareto front to evaluate the closeness, the degree and the betweenness values of all nodes, we arrive at the most central, well-connected and critical residues of the FV-structure, located at the junction of multiple domains, and most likely holding the structure in its most favorable conformation. Abbreviations: High degree and high betweenness (HDHB); low degree and high betweenness (LDHB); low degree and low betweenness (LDLB); Solvent accessible surface area (SASA). Statistics: One-way ANOVA followed by Tukey’s Post Hoc test. *** *p* value < 0.001.

After creating the FV-RIN and determining the most appropriate centrality measures to quantify the position of its residues, we aimed to group the FV amino acids according to their centrality characteristics (Supplementary Table S2); this should facilitate the analysis of the different regions of the FV protein, as well as uncover the relation between an amino acid’s position within the network and the disease that ensues upon a non-synonymous mutation. For this purpose, we elaborated a procedure that automatically identifies critical residues within 3 different criticality levels, namely, those with high degree and high betweenness (HDHB); low degree and high betweenness (LDHB); and low degree and low betweenness (LDLB). This procedure automatically divides the degree/log-betweenness space into 4 uniform bins (Fig. 2A). In the full FV-RIN, this technique yielded 63 residues classified as HDHB, 19 as LDHB and 34 LDLB (Fig. 2B). In biological terms, it means that HDHB residues are conserved, located at the core of FV, take part in multiple atomic interactions with other residues, and serve as bridges for long-range contacts between residues located far apart in the structure (Fig. 2C–D). The LDHB residues are located in an intermediate layer between the core of FV and its surface, and despite its low number of direct connections, they seem pivotal in maintaining the surface residues in place^25,26^. Finally, the LDLB residues are mainly surface residues, taking part in only a few inter-atomic interactions and not central in the protein structure organization (although they compose the protein’s binding sites^26^).

Together, these findings indicate that the degree and the betweenness can successfully assign a quantitative measure to every and each residue of FV, turning the abstract concepts of ’core’ and ’surface’ into clear numerical values that can be used for further analyses.

Next, we wondered what are the most critical residues of the whole FV protein structure. To answer this question, in addition to the degree and the betweenness, we added the closeness values to the analysis of criticality of the residues — this should quantify the importance of residues from a local (i.e. degree) as well as a global connectivity perspective (betweenness and closeness). We used the Pareto front to find which residues had the highest values in terms of degree, betweenness and closeness values (Fig. 2E); we named theses residues as *supercritical*. In the FV-RIN, 8 residues were found to meet this criteria: Met618, Phe1745, Val1814, Leu1836, Leu1837, Phe1872, Leu1873 and Ile1944. As Fig. 2B depicts, the supercritical residues are buried deep in the core and while they are relatively conserved, they are not the most conserved residues of FV, and most likely work in conjunction with other less connected but important residues to maintain the FV structure and function (Supplementary Figure S2).

From these observations, we understand that centrality measures derived from the FV-RIN are able to identify residues that exert a critical function in the FV structure. Moreover, it is reassuring to verify that the critical residues identified in the FV-RIN are located within the core of the protein and have changed very little during the course of millions of years of evolution, corroborating that the centrality measures can indeed uncover features that have biological meaning.

### Mapping of mutation data into the FV-RIN

**Figure 3:**
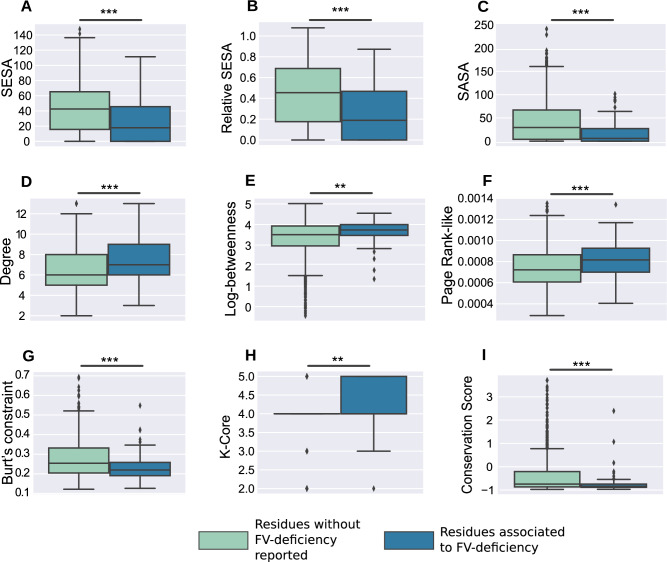
The hallmarks of residues associated to FV-deficiency. The figure depicts a comparison of structural, evolutionary and network centrality properties of the FV residues associated to FV-deficiency (n=63), compared to those without FV-deficiency reported ($n=1254$). The substitution of residues with low surface-exposed and surface accessible areas (i.e., the core residues), often lead to the loss-of-function of proteins^67^ (panels A-C). Likewise, mutations of the most central residues, as indicated by multiple centrality measures derived from the FV-RIN, are also related to the occurrence of FV-deficiency (panels D-H). Finally, in agreement with these observations, substitutions of the most conserved residues of FV are also likely to impair the functions of FV (panel I, where low values indicate high conservation). The p-values are derived from a statistical comparison designed to minimize the effect of different group sizes (i.e., the bootstrap hypothesis testing^45,46^; see Methods). *** *p* value < 0.001; ** *p* value < 0.01; * *p* value < 0.05. Abbreviations: SESA, solvent exposed surface area; SASA: Solvent accessible surface area. Supplementary Tables S2–S3 contain all measures used in the study.

Finally, we investigated what is the effect of non-synonymous amino acid substitutions in the function of FV. We manually gathered all single-point missense mutations reported in the European Association for Haemophilia and Allied Disorders database (EAHAD), and after a careful data sanitation to eliminate duplicate and ambiguous records, we consolidated a dataset with 63 unique mutations. We asked what are the structural, evolutionary and centrality characteristics of the residues where these substitutions occurred, and how they compare to other positions in the FV protein. We found a clear pattern that strongly associates the occurrence of FV-deficiency and the position where a mutation happens (Fig. 3).

Our results show that most substitution of residues located near the core of the protein lead to FV-deficiency, and this is explained by the fact that these residues have low surface exposure, are highly central in the FV-RIN, and are conserved — the hallmarks of residues that hold protein structures structure in place. Moreover, we found an overlap of ${\sim }$17% between the FV-deficiency and the central residues (i.e., the HDHB and LDHB groups). We found no overlap between the supercritical residues and the FV-deficiency residues; we hypothesize that mutations at these residues would lead to a complete non-functional FV-protein (a lethal mutation), or we do not see a larger overlap simply because the groups are too small.

Nevertheless, we listed the centrality values, structural and evolutionary features of all FVa residues, effectively building a resource that the community can examine and use for their own research (Supplementary Tables S2–S3).

In summary, these observations demonstrate the feasibility of using measures derived from the FV structure, as well as from its evolutionary history to study the basal mechanism leading to FV-deficiency. Similar to other proteins involved in the coagulation cascade^27,28^, mutations at the core residues of FV hampers its ability to participate in key interactions, most likely because its structure and correct folding are disrupted.

### Predicting the effect of new mutations

After relating the occurrence of FV-deficiency to the centrality properties of the FV-RIN and to the structural and evolutionary measures of its residues, we wondered if we could use all these features in conjunction to predict the effect of new mutations. In practice, these predictions serve two purposes; first, it could anticipate the manifestation of FV-deficiency in patients harboring new single-point, non-synonymous mutations in the F5 gene; and second, by understanding which mutations are more likely to impair FV’s function, researchers can avoid these substitutions when performing targeted mutations aiming to improve FV’s activity, stability and half-life. In fact, as we did for Coagulation factors VIII and IX (FIX) (refs.^27–29^), these predictions create a comprehensive map of the protein regions that are harmful or safe to substitute.

**Figure 4:**
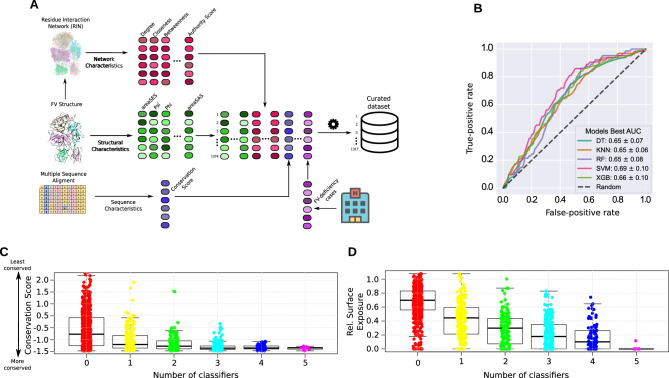
The FV-Class AI framework. (**A**) The first and most critical step to use predictive ML algorithms is to prepare a clean, highly informative dataset. We used the structural characteristics of the FV structure^15^ (PDB: 7KVE), as well as centrality measures derived from the FV residue network, and a score quantifying the evolutionary conservation of its residues. In this dataset, we had 63 unique instances representing single-point mutations of FV-deficient patients and ${\sim }$1250 residues where no FV-deficiency was reported (Supplementary Table S4). After careful curation and standardization, this dataset was ready to be inputted into multiple ML classifier algorithms. (**B**) We performed a comprehensive parameter optimization to find the best settings for the FV-Class; this yielded AUC values that demonstrate a favorable learning prospect for all algorithms (Methods). In particular, the Support Vector Machine (SVM) obtained the highest value. (**C–D**) Here, each dot is a residue of the FV protein, and the boxplots depict the number of ML classifiers that predicted a loss-of-function if those residues are mutated; for instance, in red are the residues predicted to be safe to substitute, and in magenta, those that will most likely impair FV’s functions (in general, the most conserved residues, buried at the core of the structure). Abbreviations: DT: Decision trees^32^; KNN: K-nearest neighbors^36^; RF: Random forest^36^; XGB: XGBoost^34^.

For this purpose, we devised a full-scale machine learning (ML) framework with different underlying classifier algorithms and training regimens — we named this framework FV-Class (Fig. 4A). A ML classifier algorithm work by tuning its parameters in a way that allows the classifier to learn intrinsic data patterns from a set of examples (the training set). After the algorithms converge (i.e., achieve good accuracy on the training set), its real performance is then assessed on a set of examples not seen during the training phase (the so-called test-set). In our case, we prepared a dataset where the features were the network centrality measures, the structural and evolutionary characteristics of FV, and the class label to be predicted was the presence or absence of FV-deficiency. This dataset presented two challenges that are notorious in the ML field^30^, first, a small number of examples, and second, imbalanced classes (i.e, 63 FV-deficiency against 1254 non-FV-deficiency cases).

We addressed this issue by implementing the FV-Class using a series of pre-processing steps and careful training routines (see Methods). In brief, we varied both the pre-processing routine as well as the estimator used to train the model. In all cases, we standardized the features (null mean and unit standard deviation) to eliminate biases induced by data scales. For the pre-processing, we considered 4 different combinations: using PCA or not for dimensionality reduction, and using oversampling or not for balancing the classes. The ADASYN^31^ strategy was used to oversample the minority class until balance was achieved. For the estimators, we used the following classifiers: Decision Tree^32^; Random Forest^33^; Extreme Gradient Boosting (XGBoost)^34^; Support Vector Machine (SVM)^35^ and k Nearest Neighbors (kNN)^36^. To tune the estimators’ hyperparameters, we first employed a Grid-Search strategy with 10-fold stratified cross-validation (given the target imbalance). The mean validation Area Under the Receiver Operating Characteristics Curve (AUC) was used as criterion for choosing the best set of hyperparameters (see Methods).

In the AUC, a value of 0.5 indicates a random prediction, while a value of 1.0 represents a perfect classification. In our case, after a comprehensive grid-search procedure, we found that our models achieved fair AUC values in the range of 0.65-0.69, suggesting that while the results do not reach the necessary level required for clinical FV-deficiency diagnosis, the classifiers learned the intrinsic features of the data (Fig. 4B). Moreover, we also used an existing program^37^ (Polyphen-2) to anticipate the effect of single-point, non-synonmous mutations, however, its output scores could not distinguish the different phenotypes caused by FV-deficiency (Supplementary Figure S3). We also devised an alternative ML framework with a different training regimen and that uses both supervised and unsupervised algorithms, but the AUC was not superior to the current FV-Class setup, highlighting the difficulty in fully anticipating the occurrence of this condition (Supplementary Results). Nevertheless, we regard as encouraging the fact that the FV-Class algorithms achieved this AUC value despite the dramatic limitations in the size of the input dataset. For this reason, we implemented the FV-Class framework in a way that it is straightforward to be retrained as soon as new clinical and genetic data becomes available.

Finally, we used the current FV-Class framework to anticipate the effect of mutations on residues not listed in any database. This exercise is particularly interesting to aid in the generation of recombinant FV proteins, because it can dramatically reduce the number of candidates to be validated in in vitro and in vivo assays. For this purpose, we used all ML classifiers in conjunction, and counted the number of classifiers that predicted mutations to a given residue to be detrimental to FV’s functions (Fig. 4C–D).

To achieve this goal, we utilized 5 ML classifiers in conjunction to predict the chances of a substitution being detrimental to FV’s function. Our findings revealed that ${\sim }$40% of residues were deemed safe to substitute — i.e., they were predicted by all 5 classifiers as having low probability of causing harmful effects, possibly due to favorable structural, evolutionary and network centrality characteristics. Additionally, we found that 33% of residues presented an intermediate prospect of being harmful, and less than 1% of residues were classified as detrimental by all algorithms (the most conserved and buried residues of FV). We assigned a quantitative score to each residue, effectively ranking them based on their potential to disrupt FV’s activity (Supplementary Table S5); if considered in conjunction with other factors like the structural, evolutionary, and centrality measures, these results reveal the most promising regions for targeted mutations.

In summary, these findings demonstrate that using multiple features derived from the FV protein is a powerful strategy to anticipate the risk of FV-deficiency in patients harboring non-synonymous mutations. Moreover, it is interesting that the FV-Class identified regions of the FV protein that are more vulnerable to amino acid substitutions — and these findings are in agreement with the common knowledge about the role of the different parts of the protein architecture.

## Discussion

In this study, we created a comprehensive map of the FV protein structure, effectively quantifying the importance of its residues and associating their substitution to the occurrence of FV-deficency. We created a residue network where the residues of FV are connected if they are in close proximity in the three-dimensional space; we used multiple algorithms to quantify the position of each residue within this network, and observed that the hub residues — i.e., those conserved and located at the core of the structure — are usually associated to loss of function if mutated to a different amino acid. Finally, we established a machine learning framework (the FV-Class) that took as input multiple features from FV and single-point non-synonymous substitutions from FV-deficiency patients, and anticipated with reasonable accuracy the effects of novel mutations in this protein.

The idea of transforming a protein structure into an undirected graph has been explored previously and has proven itself to be a powerful method to quantify the importance of the thousands of residues (Fig. 1). In our previous studies, we found a strong association between the measures derived from residue networks and the loss-of-function of the FVIII, FIX and Antithrombin (AT) proteins^27,28,38^. Likewise, we found that residues associated to FV-deficiency are conserved, buried at the core and take part in multiple inter-atomic interactions (Fig. 3). This impairment of function is an underlying property of different network systems, including electricity grids^39^ and transportation networks^40^, where perturbations to the most connected nodes disrupt the layout and communication of the whole system. As we observed for FV, this seems to be the case, and while current datasets do not have enough resolution to anticipate precisely which function will be disrupted, the emerging picture indicates that substitutions of the most central residues impairs FV’s activity.

Compared to our previous studies^27,28^, the present work was by far the most challenging, given the rarity of FV-deficiency. In turn, this required us to use more strict statistical procedures and to develop a robust machine learning framework. In particular, the current FV-deficiency dataset had two notorious characteristics known to impair classifier algorithms’ performance^30^: imbalanced classes and a small sample size in one of the classes (63 FV-deficiency cases vs 1254 non-deficiency residues). Nevertheless, the FV-Class still achieved an AUC value of 0.69, indicating that its underlying algorithms were learning the patterns that relate the characteristics of residues in the FV protein to its loss-of-function. Importantly, we designed this framework as an open-source tool that can be used to study other diseases, and be retrained as soon as new FV-deficiency cases are reported in the biomedical literature.

While the FV-Class and the FV-RIN are not yet suitable for routine diagnosis in clinical settings, we still regard it as useful for researchers interested in generating recombinant FV molecules that display enhanced activity and stability. For instance, the FV map that we created (Supplementary Table S5), serves as a valuable resource to eliminate recombinant candidates that substitute residues with high centrality measures and that are evolutionary conserved, given that they are likely to lead to non-functional proteins (Fig. 4C–D). Therefore, while it is not feasible at present for any computational tool to anticipate mutations that are beneficial^41^, the FV-Class helps reduce the number of candidate molecules that will be tested in wet-lab experiments.

Finally, the present study opens interesting research avenues. First, the use of blood marker parameters to enhance the predictive power of ML algorithms. Parameters like D-dimer, aPTT and levels of circulating coagulation factors are known to play a major role in the outcome of coagulation disorders^42,43^; hence, adding them to the the input of the FV-Class is likely to enhance its capacity. Second, isolate the pro- and anti-coagulant functions of FV. As this protein exerts multiple roles depending on the stage of its life-cycle^6–8^, studying separately the network centrality and the role of its binding sites might uncover the aspects of the FV biology responsible for its opposing functions. Moreover, although our FV-RIN was built using a single conformation of the FV structure, proteins exist in multiple possible conformations (i.e., the ensembles). These conformations dynamically change the landscape of atomic interactions in the structure^44^. Computational molecular dynamics techniques can sample from conformational ensembles and build multiple RINs that might reveal which residue interactions are stable or only transient. Although not explored in this study, this remains an exciting approach to strengthen the datasets used as input to the ML algorithms. Third, not all mutations happen on coding regions, hence, adding information about the promoter regions, RNA folding and splicing sites are features that albeit challenging to represent, are certainly exciting research prospects.

In summary, by studying the FV protein from a computational perspective and using the knowledge of FV-deficiency cases accumulated over the years, we uncovered patterns of this critical component of the coagulation system. By quantifying and ranking residues according to their importance, we feel confident that the research community will generate sound hypotheses that can be tested using a fraction of the resources otherwise needed.

## Methods

### Statistical analyses

To compare the structural, evolutionary and network measures from Fig. 3, we used a statistical method less influenced by large differences in sample sizes. As a form of bootstrap hypothesis testing^45,46^, for each centrality measure separately, we randomly selected from the larger group (”No deficiency reported, $n=1254$), the same number of elements as the smaller group (FV-deficiency, $n=63$), and compared their mean values. We repeated this procedure 10,000 times and the derived p-values were the number of times that the median of the larger group was greater than the median of the FV-deficient group, divided by 10,000.

### Calculation of the FVIII protein structure properties

We used the FV protein structure deposited in the PDB with the accession code 7KVE (ref.^15^), and performed a side-chain readjustment (also known as “relax”) using Rosetta^47^. We selected the model with the lowest free energy for further analyses, and used Chimera version 1.15 (ref.^48^) to calculate and extract the following residues’ structural properties: kdHydrophobicity, solvent accessible and solvent-excluded surface areas, dihedral angles phi and psi, and the relative SESA, which was calculated by dividing the solvent-excluded area of the residue by the surface area of the same type of residue in a reference state (we considered the reference values of the 20 standard amino acids in Gly-X-Gly tripeptides)^49^.

### The FVIII residue interaction network

The FV structure was represented as an undirected unweighted graph constructed using RINerator version 0.5.1^16^ with the default parameters. We considered two residues as interacting partners if there was an edge between them in the graph. The RIN was analyzed with the Python iGraph^50^ package (version 0.9.6). The graph was simplified to remove redundant edges and self interactions. We calculated the following centrality measures: degree, betweenness, closeness, KCore, Burt’s constraint, Authority Score and Page Rank-like score. Cytoscape version 3.8.2^51^ was used to visualize the RIN. The conservation score was obtained from the ConsurfDB webserver^52^, using the FV protein structure as input.

### Construction of the machine learning framework

In supervised learning, we are interested in producing a model given by the hypothesis function $f: \mathscr{X} \rightarrow \mathscr{Y}$ which better relates the features (in the feature space ${\mathscr{X} }$) to the target (in the target space $\mathscr{Y}$), based on the available dataset $(x_i, y_i)_{i=1}^N$. In our case, we used as features: the residues’ structural properties (given by Chimera^48^); the centrality measures (given by iGraph^50^) and the conservation score (given by the ConsurfDB webserver^52^), totaling 14 features. The target was binary: 1 if a mutation in a given residue position in FV was observed, and 0 otherwise. We had $N=1317$ instances, and the target was heavily imbalanced: 1254 ($95.22 \%$) observations in class 0 and 63 ($4.78 \%$) in class 1. Fifty-seven instances were discarded because they had missing values due to problems in the underlying 3D structure model (i.e., errors in the Cryo-EM structure determination), or the value 0 in the betweenness centrality.

All the main modeling tools were used from the Python^53^ package scikit-learn^54^ (version 1.0.2) and imbalanced-learn^55^ (version 0.9.0).

For the estimators, the following classification methods were used: Decision Tree^32^; Random Forest^33^; Extreme Gradient Boosting (XGBoost)^34^; Support Vector Machine (SVM)^35^ and k-Nearest Neighbors (kNN)^36^. To tune the estimators’ hyperparameters, we first employed a Grid-Search strategy with 10-fold stratified cross-validation (given the target imbalance). The mean validation AUC was used as criterion for choosing the best set of hyperparameters. The hyperparameter grids tested for each one of the estimators were the following (all the unspecified hyperparameters were left as their default values in the scikit-learn implementation):

For the Decision Tree, we varied the splitting criteria (Gini impurity or entropy), the minimum number of samples in a node for a split to be considered (varied from 2 to 50), the minimum number of samples in a node for it to be considered a leaf (varied from 1 to 20), and the cost-complexity pruning^56^ parameter (varied in the range (1, 0.1, 0.01, 0.001, 0.0001)).

For the Random Forest, we varied the number of trees in the ensemble (from 50 to 1500, with a step of 50), the number of features in the random subset of features considered as candidates for each split (from 2 to 7), and the minimum number of samples in a node for it to be considered a leaf (varied from 1 to 10);

For the XGBoost, we varied the maximum depth of the trees in the ensemble (varied from 1 to 25), the L2 regularization weight (varied in the range (1, 0.1, 0.01, 0.001, 0.0001)) and the learning rate (in the range (1, 0.1, 0.01, 0.001, 0.0001)).

For the SVM, we varied the kernel function (polynomial or Radial Basis Function), the $\gamma$ kernel coefficient (varied from 0.01 to 1.5, with a step of 0.05), the degree of the polynomial kernel (varied from 2 to 5), the independent term in the polynomial kernel (varied from 0.1 to 2, with a step of 0.05).

For the kNN, the only hyperparameter varied was the number of neighbors considered in the classification (from 3 to 50).

The classification metrics (AUC) presented for each one of the tuned classifiers were calculated with 10-fold stratified cross-validation. The mean and standard deviation of the metrics were reported.

We also employed Bayesian optimization with the library Hyperopt^57^ (version 0.2.7) as an alternative strategy for optimizing the estimators’ hyperparameters. Hyperopt’s implementation uses Tree of Parzen Estimators (TPE)^58^ as surrogate model. We did 50 evaluations over an extended hyperparameter space for each one of the estimators.

### Programming language and packages

To create the graph using the RINerator software, we used Python 2.7.18^59^ with the NumPy^60^ (version 1.16.6) and Biopython^61^ (version 1.59) packages. The other calculations, figures and analysis were done with Python 3.9.9^53^ using the following packages: Hydra^62^ (version 1.1.1), Pandas^63^ (1.3.5), NumPy^60^ (version 1.21.4), Igraph^50^ (version 0.9.6), Scikit-Learn^54^ (version 1.0.2), Imblearn^55^ (version 0.9.0), XGBoost^34^ (version 1.5.2), Seaborn^64^ (version 0.12.1) and Matplotlib^65^ (version 3.6.0). We prepared a DockerFile^66^ to facilitate the instalation and reproduction of the results of this study.

## Supplementary Information

Supplementary Information 1.

Supplementary Information 2.

## Data Availability

The source code and datasets used in the study are freely available at https://github.com/madlopes/FV-Class.

## References

[1] Lee, C. A., Berntorp, E. E. & Hoots, W. K. *Textbook of hemophilia* (John Wiley & Sons, 2011).

[2] Jenny RJ (1987). Complete cdna and derived amino acid sequence of human factor v. Proc. Natl. Acad. Sci..

[3] Kane WH, Majerus PW (1981). Purification and characterization of human coagulation factor v. J. Biol. Chem..

[4] Stormorken H (2003). The discovery of factor v: A tricky clotting factor. J. Thromb. Haemost..

[5] Hayward CP (1995). Factor v is complexed with multimerin in resting platelet lysates and colocalizes with multimerin in platelet $$\alpha$$-granules. J. Biol. Chem..

[6] Camire RM (2011). A new look at blood coagulation factor v. Curr. Opin. Hematol..

[7] Yang TL (1998). The structure and function of murine factor v and its inactivation by protein c. Blood J. Am. Soc. Hematol..

[8] Mann KG, Kalafatis M (2003). Factor v: A combination of dr jekyll and mr hyde. Blood J. Am. Soc. Hematol..

[9] Asselta R, Tenchini M, Duga S (2006). Inherited defects of coagulation factor v: The hemorrhagic side. J. Thromb. Haemost..

[10] Gonzalez-Boullosa R (2005). The use of activated recombinant coagulation factor vii during haemarthroses and synovectomy in a patient with congenital severe factor v deficiency. Haemophilia.

[11] Petros S, Fischer J, Mössner J, Schiefke I, Teich N (2008). Treatment of massive cecal bleeding in a 28-year-old patient with homozygous factor v deficiency with activated factor vii. Z. Gastroenterol..

[12] Chediak J, Ashenhurst JB, Garlick I, Desser RK (1980). Successful management of bleeding in a patient with factor v inhibitor by platelet transfusions. Blood.

[13] Cimini G (2019). The statistical physics of real-world networks. Nat. Rev. Phys..

[14] Koutrouli, M., Karatzas, E., Paez-Espino, D. & Pavlopoulos, G. A. A guide to conquer the biological network era using graph theory. *Front. bioeng. biotechnol.* 34 (2020). 10.3389/fbioe.2020.00034 PMC7004966 32083072

[15] Ruben EA, Rau MJ, Fitzpatrick JA, Di Cera E (2021). Cryo-em structures of human coagulation factors v and va. Blood J. Am. Soc. Hematol..

[16] Doncheva NT, Klein K, Domingues FS, Albrecht M (2011). Analyzing and visualizing residue networks of protein structures. Trends Biochem. Sci..

[17] Dokholyan NV, Li L, Ding F, Shakhnovich EI (2002). Topological determinants of protein folding. Proc. Nat. Acad. Sci..

[18] Reichmann D (2005). The modular architecture of protein-protein binding interfaces. Proc. Nat. Acad. Sci..

[19] Nisthal A, Wang CY, Ary ML, Mayo SL (2019). Protein stability engineering insights revealed by domain-wide comprehensive mutagenesis. Proc. Nat. Acad. Sci..

[20] Gerasimavicius L, Liu X, Marsh JA (2020). Identification of pathogenic missense mutations using protein stability predictors. Sci. Rep..

[21] Yan W (2014). The construction of an amino acid network for understanding protein structure and function. Amino Acids.

[22] Censoni, L., dos Santos Muniz, H. & Martínez, L. A network model predicts the intensity of residue-protein thermal coupling. *Bioinformatics* **33**, 2106–2113 (2017). 10.1093/bioinformatics/btx124 28334219

[23] Amitai G (2004). Network analysis of protein structures identifies functional residues. J. Mol. Biol..

[24] del Sol A, Fujihashi H, Amoros D, Nussinov R (2006). Residue centrality, functionally important residues, and active site shape: analysis of enzyme and non-enzyme families. Protein Sci..

[25] Dokholyan NV, Li L, Ding F, Shakhnovich EI (2002). Topological determinants of protein folding. Proc. Natl. Acad. Sci..

[26] Reichmann D (2005). The modular architecture of protein-protein binding interfaces. Proc. Natl. Acad. Sci..

[27] Lopes TJ, Rios R, Nogueira T, Mello RF (2021). Prediction of hemophilia a severity using a small-input machine-learning framework. NPJ Syst. Biol Appl..

[28] Lopes TJ, Nogueira T, Rios R (2022). A machine learning framework predicts the clinical severity of hemophilia b caused by point-mutations. Front. Bioinformat..

[29] Lopes TJ, Rios R, Nogueira T, Mello RF (2021). Protein residue network analysis reveals fundamental properties of the human coagulation factor viii. Sci. Rep..

[30] Hastie, T., Tibshirani, R., Friedman, J. H. & Friedman, J. H. *The elements of statistical learning: data mining, inference, and prediction*, vol. 2 (Springer, 2009).

[31] He, H., Bai, Y., Garcia, E. A. & Li, S. Adasyn: Adaptive synthetic sampling approach for imbalanced learning. In *2008 IEEE international joint conference on neural networks (IEEE world congress on computational intelligence)*, 1322–1328 (IEEE, 2008).

[32] Quinlan JR (1986). Induction of decision trees.. Mach. Learn..

[33] Breiman L (2001). Random forests. Mach. Learn..

[34] Chen, T. & Guestrin, C. XGBoost: A scalable tree boosting system. In *Proceedings of the 22nd ACM SIGKDD International Conference on Knowledge Discovery and Data Mining*, KDD ’16, 785–794, 10.1145/2939672.2939785 (ACM, New York, NY, USA, 2016).

[35] Cortes C, Vapnik V (1995). Support-vector networks.. Mach. Learn..

[36] Fix, E. & Hodges, J. L. Discriminatory analysis. nonparametric discrimination: Consistency properties. *Int. Stat. Rev/Rev. Int. de Stat.* **57**, 238–247 (1989).

[37] Adzhubei IA (2010). A method and server for predicting damaging missense mutations. Nat. Methods.

[38] Lopes TJ (2023). Computational analyses reveal fundamental properties of the at structure related to thrombosis. Bioinf. Adv..

[39] Arianos, S., Bompard, E., Carbone, A. & Xue, F. Power grid vulnerability: A complex network approach. *Chaos Interdiscip. J. Nonlinear Sci.* **19**, 013119 (2009). 10.1063/1.3077229 19334983

[40] Xu Z, Harriss R (2008). Exploring the structure of the us intercity passenger air transportation network: A weighted complex network approach. GeoJournal.

[41] Broom A, Trainor K, Jacobi Z, Meiering EM (2020). Computational modeling of protein stability: Quantitative analysis reveals solutions to pervasive problems. Structure.

[42] Provan, D. & Gribben, J. *Molecular hematology* (John Wiley & Sons, 2010).

[43] Dunn, A. L., Kerlin, B. A., O’Brien, S. H., Rose, M. J. & Kumar, R. *Pediatric bleeding disorders: A clinical casebook* (Springer, 2020).

[44] Bonomi M, Heller GT, Camilloni C, Vendruscolo M (2017). Principles of protein structural ensemble determination. Curr. Opin. Struct. Biol..

[45] Johnson RW (2001). An introduction to the bootstrap. Teach. Stat..

[46] Davison, A. C. & Hinkley, D. V. *Bootstrap methods and their application*. 1 (Cambridge university press, 1997).

[47] Leaver-Fay A (2011). Rosetta3: An object-oriented software suite for the simulation and design of macromolecules. Method Enzymol..

[48] Pettersen EF (2004). Ucsf chimera-a visualization system for exploratory research and analysis. J. Comput. Chem..

[49] Bendell CJ (2014). Transient protein-protein interface prediction: datasets, features, algorithms, and the rad-t predictor. BMC Bioinformatics.

[50] Csardi, G. & Nepusz, T. The igraph software package for complex network research. *InterJ.* **Complex Syst.**, 1695 (2006).

[51] Shannon P (2003). Cytoscape: A software environment for integrated models of biomolecular interaction networks. Genome Res..

[52] Ashkenazy H (2016). Consurf 2016: An improved methodology to estimate and visualize evolutionary conservation in macromolecules. Nucleic Acids Res..

[53] Van Rossum G, Drake FL (2009). Python 3 Reference Manual.

[54] Pedregosa F (2011). Scikit-learn: Machine learning in Python. J. Mach. Learn. Res..

[55] Lemaître G, Nogueira F, Aridas CK (2017). Imbalanced-learn: A python toolbox to tackle the curse of imbalanced datasets in machine learning. J. Mach. Learn. Res..

[56] Breiman, L., Friedman, J. H., Olshen, R. A. & Stone, C. J. *Classification and regression trees* (Routledge, 2017).

[57] Bergstra, J., Yamins, D. & Cox, D. Making a science of model search: Hyperparameter optimization in hundreds of dimensions for vision architectures. In *International conference on machine learning*, 115–123 (PMLR, 2013).

[58] Bergstra, J., Bardenet, R., Bengio, Y. & Kégl, B. Algorithms for hyper-parameter optimization. *Adv. Neural inf. Process. Syst.* **24** (2011).

[59] Van Rossum, G. & Drake Jr, F. L. *Python reference manual* (Centrum voor Wiskunde en Informatica Amsterdam, 1995).

[60] Harris CR (2020). Array programming with NumPy. Nature.

[61] Cock, P. J. A. *et al.* Biopython: freely available Python tools for computational molecular biology and bioinformatics. *Bioinformatics* **25**, 1422–1423, 10.1093/bioinformatics/btp163 (2009). https://academic.oup.com/bioinformatics/article-pdf/25/11/1422/944180/btp163.pdf. 10.1093/bioinformatics/btp163 PMC2682512 19304878

[62] Yadan, O. Hydra - a framework for elegantly configuring complex applications. Github (2019).

[63] Wes McKinney. Data structures for statistical computing in python. In Stéfan van der Walt & Jarrod Millman (eds.) *Proceedings of the 9th Python in Science Conference*, 56 – 61, 10.25080/Majora-92bf1922-00a (2010).

[64] Waskom, M. L. Seaborn: Statistical data visualization. *J. Open Sour. Softw.* **6**, 3021, 10.21105/joss.03021 (2021).

[65] Hunter JD (2007). Matplotlib: A 2d graphics environment. Comput. Sci. Eng..

[66] Merkel D (2014). Docker: Lightweight linux containers for consistent development and deployment. Linux J..

[67] Whitford, D. *Proteins: structure and function* (John Wiley & Sons, 2013).

