# Supplementary material for: Full-scale network analysis reveals properties of the FV protein structure organization
Source: Sci Rep. 2023 Jun 12;13:9546. doi: 10.1038/s41598-023-36528-z (PMC10260930; doi:10.1038/s41598-023-36528-z)
Supplement: Supplementary file 1 — Supplementary Information 1. [file 41598_2023_36528_MOESM1_ESM.pdf]

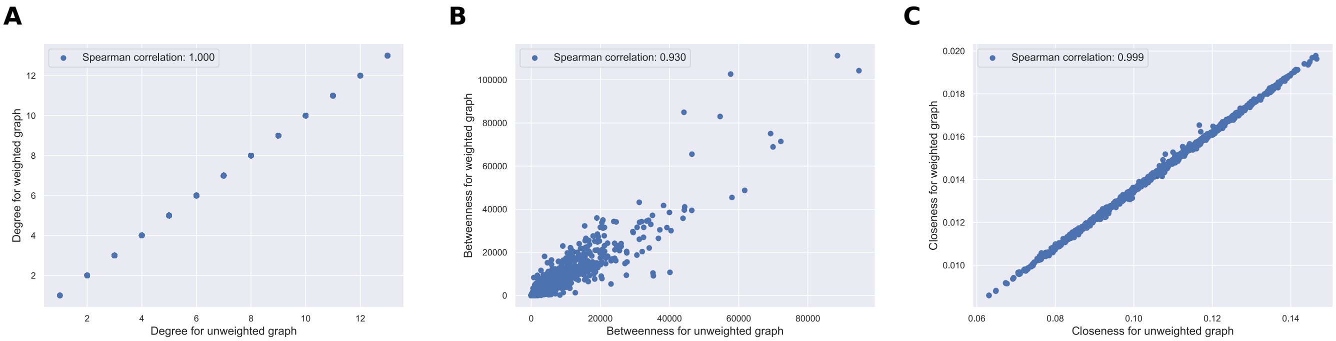

Supplementary Figure 1: RIN construction with weighted edges.
(A-C) The figure depicts the Spearman correlation between the centrality measures derived from the FV-RIN upon building it as an *undirected weighted graph* versus an *undirected unweighted graph*. As expected, the degree has perfect correlation, as it does not depend on the weights of the edges. The betweenness and the closeness centralities have only small variation, leading to a very strong correlation between the values derived from both networks.

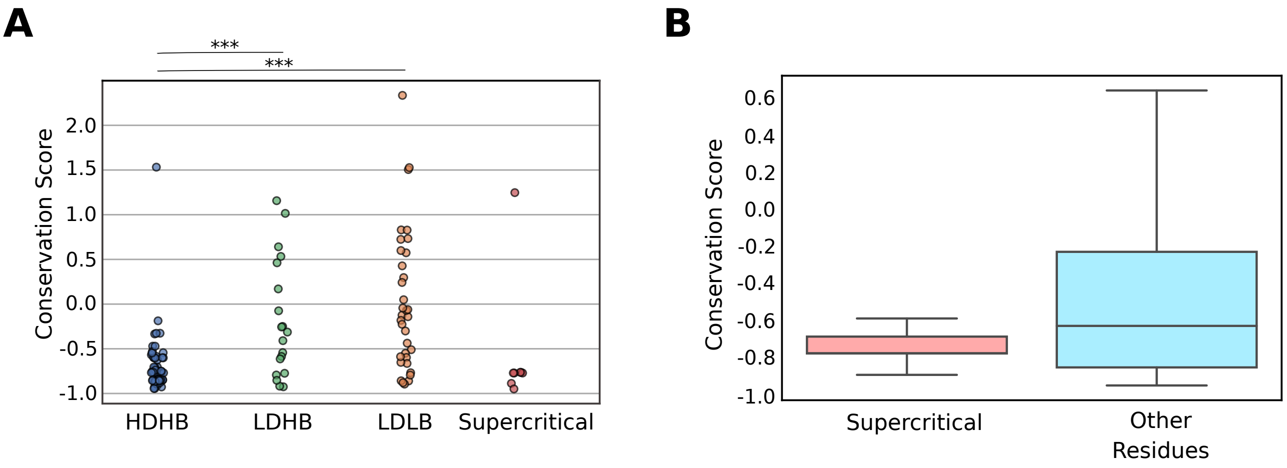

Supplementary Figure 2: Assessment of FV residues' conservation.

(A) FV residues located at the core of the structure tend to be more conserved than residues located closer to the surface (lower scores indicate higher conservation). (B) Although the supercritical residues are among the most conserved residues of the structure, they are not the most conserved, despite their highly-central position and inter-atomic interaction with several other residues. Statistics: Panel A, One-way ANOVA followed by Tukey's Post Hoc test; panel B, bootstrap hypothesis testing (see Methods). *** p-value < 0.001.

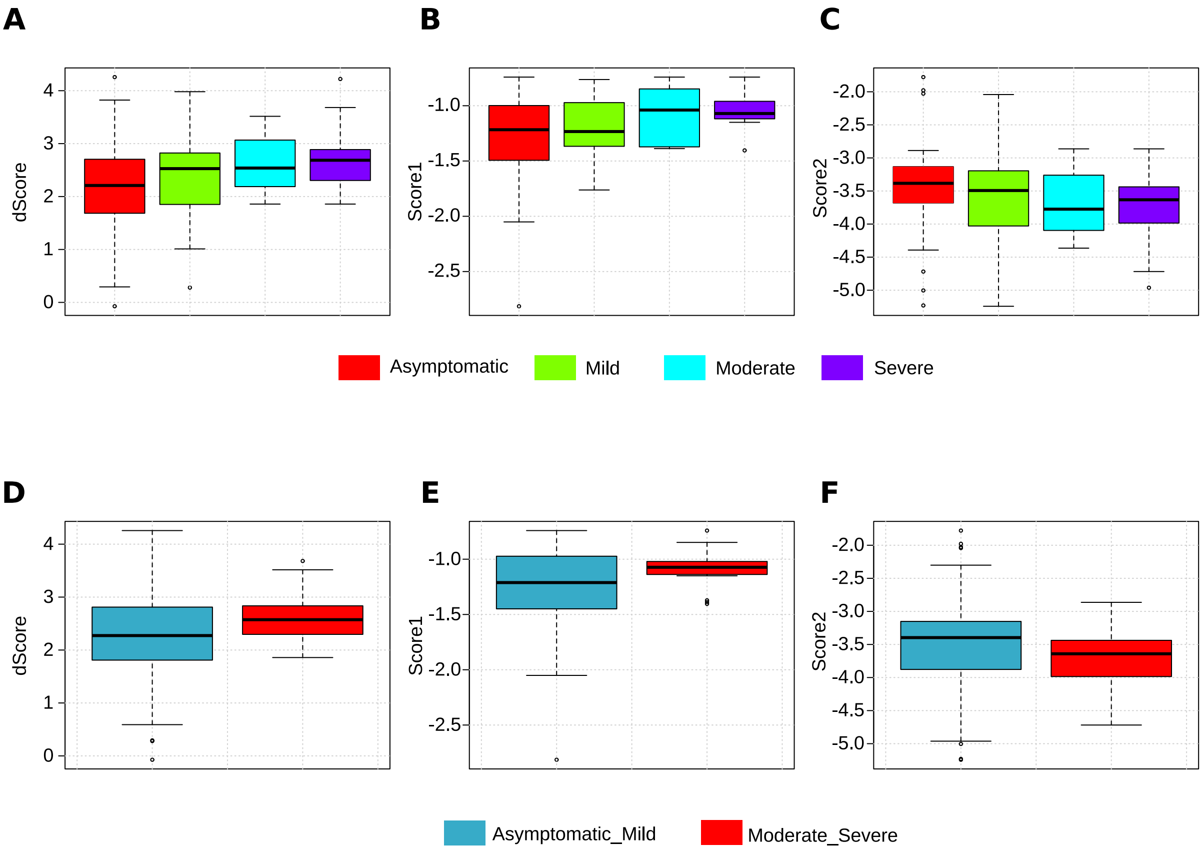

Supplementary Figure 3: Use of existing programs to distinguish harmful mutations
Polyphen-2 generate scores that can be used to distinguish, to a modest extent, the effects of mutations in the FV protein (Adzhubei et al., 2013). The boxplots depict the median (centerline), the first and third quartiles (lower- and upper-bounds), and 1.5 times the inter-quartile range (lower- and upper whiskers). We used the two-tailed unpaired Wilcoxon test, but no significant differences were found neither for the reported or the merged severities of FV-deficiency.

Supplementary Results - Full-Scale Network Analysis Reveals Properties of the FV Protein Structure Organization

André J. Ferreira–Martins[a], Rodrigo Castaldoni[a], Brenno M. Alencar[b], Marcos V. Ferreira[b], Tatiane Nogueira[b], Ricardo A. Rios[b], Tiago J.S. Lopes[c]

[a]*Center for Advanced Analytics, Itaú Unibanco, Praca Alfredo Egydio de Souza Aranha, 100, Parque Jabaquara, São Paulo, 04344-902, SP, Brazil*
[b]*Institute of Computer Science, Federal University of Bahia, Avenida Milton Santos, Ondina, Salvador, 40.170-110, BA, Brazil*
[c]*Center for Regenerative Medicine, National Centre for Child Health and Development Research Institute, 2-10-1 Okura,Setagaya-ku, Tokyo, 157-8535, Japan*

1. Machine Learning Approach

In this study, we used machine learning (ML) to create an intelligent program capable of identifying patterns in patients' data and predicting hemophilia caused by single-point, non-synonymous mutations in the coagulation factor V protein (FV). For this purpose, the patients' data were organized into an attribute-value matrix ($\mathbf{X}_{m \times n}$), in which a given patient $x_i \in \mathbf{X}, i <= m$ is composed of a set of n attributes $A = \{a_1, a_2, ..., a_n\}$. In our context, the attributes were extracted from the protein structures (Methods). Moreover, we separated the occurrence or non-occurrence of FV-deficiency of all patients into a vector $\mathbf{Y}$, referred to as labels, such that $|\mathbf{Y}| = m$.

Aiming to proceed with the learning process, we selected a set of ML methods from two main paradigms: supervised and unsupervised. Before discussing the application of those methods, it is worth mentioning that the patient's data ($\mathbf{X}$), formally referred to as input space, were pre-processed to remove missing values and prepare them to be modeled by the ML methods. We also evaluated methods to normalize and balance the number of examples for every expected label $\mathbf{Y}$, such as oversampling methods based on SMOTE [1]. It is worth emphasizing, though, that the best results in our experiments were obtained without applying such methods. Moreover, we designed our experiments by randomly splitting the data into training

(85%) and test (15%) folds. This proportion was selected after collecting the best results varying the test ratio from 10% to 25%, as often used in ML projects. All variations of test folds contained samples of patients respecting a balanced distribution of classes. Aiming to avoid biased conclusions, the training and test segmentation was randomly repeated 100 times, and the final results were discussed in terms of means and standard deviations. During the training phase, the search for the optimal model configurations was induced by combining the stratified 5-fold cross-validation approach and the Random Search strategy, which is a hyper-parameter optimizer implemented to find models by effectively searching a larger configuration space that even includes less promising possibilities [2]. We also considered the Grid and Bayesian Search strategies; however, we adopted the Random Search due to its computational performance and statistically similar results.

Supervised ML methods are seen as functions (f) responsible for mapping (modeling) an input space to labels $\mathbf{Y}$ as the relation $f : \mathbf{X} \rightarrow \mathbf{Y}$. Among the supervised methods assessed in this work, we only present those with the best results: K-Nearest Neighbors (KNN) [3, 4], Decision Trees (DT) [3, 5], XGBoost [6], Random Forest (RF) [6], Support Vector Machine (SVM) [7], Artificial Neural Networks (ANN) [8] and Logistic Regression (LR) [9]. However, results with SVM, ANN, and LR were omitted for presenting the worst performances.

We also assessed the performance of unsupervised methods, where the main difference is the absence of labels $\mathbf{Y}$ to support the training phase. In summary, such methods only analyze the input space searching for a partition Π that contains groups of patients with similar behavior. Given that our dataset is imbalanced, in theory unsupervised methods could present better results, especially those designed to find outliers and anomalies. In our experiments, we considered the following methods: Hierarchical Clustering (HC) [10], DBSCAN [11], Isolation Forest [12], Local Outlier Factor (LOF) [13], and One-Class SVM [14]. Results with HC, DBSCAN, and One-Class SVM were also omitted due to their poor overall performances.

The KNN model was adjusted varying the number of neighbors from 1 to 25, and the Minkowski distance from 1 to 4. Moreover, we also considered the Distance-Weighted Nearest Neighbors (DWNN) [3, 4] by weighting the class votes according to the neighbor distances. The DT model was estimated by varying the minimum number of observations in a terminal node (leaf) and the maximum depth within the interval $[1, 25]$. We also evaluated two different functions to measure node importance: Gini and Entropy. Next, we

evaluated two ensemble methods based on DT. XGBoost was estimated by varying the maximum depth of a tree inside the interval $[1, 25]$, the learning rate by scaling the contribution of each tree as $\eta \in [0.05, 0.30]$, the minimum sum of instance weight in a child in the interval $[1, 7]$, the minimum loss reduction to split a leaf node between $[0.0, 0.4]$, and the subsample ratio of columns inside the interval $[0.3, 0.7]$. The RF ensemble was trained by using the maximum depth within the interval $[1, 25]$, and the number of trees between 1 and 25. By considering those methods with the best results, we also built a Stacking ensemble with different configurations of base and meta models. However, the results were also omitted for not improving the performance compared to individual applications.

The Isolation Forest was trained by varying the number of base models from 10 to 100, the proportion of outliers in the dataset between 0.1 and 0.5, the number of patients' features from 2 to the maximum, and the presence/absence of bootstrap (training data created sampled with replacement). Local Outlier Factor was estimated by considering the number of neighbors from 5 to 45, the proportion of outliers in the dataset between 0.35 and 0.5, and the Minkowski distance from 1 to 5. All hyper-parameter values were empirically defined by analyzing every model according to the extrema points limiting the general performance, i.e., the minimum and maximum values were defined when the performance significantly dropped.

In our ML pipeline, the last step was achieved by assessing the best models on the test folds. The validation methods considered in our experiments were Accuracy (ACC) for each class, the F1 score, and the Area Under the ROC Curve (AUC) (Supplementary Figure 4). Accuracy is the most basic validation metric in ML, working as a ratio between correct classifications and all test instances. However, in our analyses, we individually analyzed the accuracy for each class. F1 works as a harmonic mean of precision and recall (a.k.a sensitivity and True Positive Rates - TPR), which are two important measures to assess the models' exactitude and completeness. In summary, they compute the rate of correct classifications for the positive label over: the number of outcomes classified as positives (precision); and the number of elements expected to be under the positive label (recall). On the other hand, AUC is based on a curve created between TPR and False Positive Rates (FPR), which is also known as specificity and computes the rate of correct classifications for the negative label over all the numbers of outcomes classified as negatives. As the area under the curve approaches 1.0, the better the final classification [15]. The F1 score was used not only to assess the

model performances on the test folds but also to induce the estimated models during the training phase. Finally, we emphasize that F1 and AUC were also considered to assess models from unsupervised approaches by considering the labels as expected partitions. In our experiments, all codes were written in Python version 3.9.12 and scikit-learn version 1.0.2 to build up the ML models, training tasks, and validation methods.

2. Results

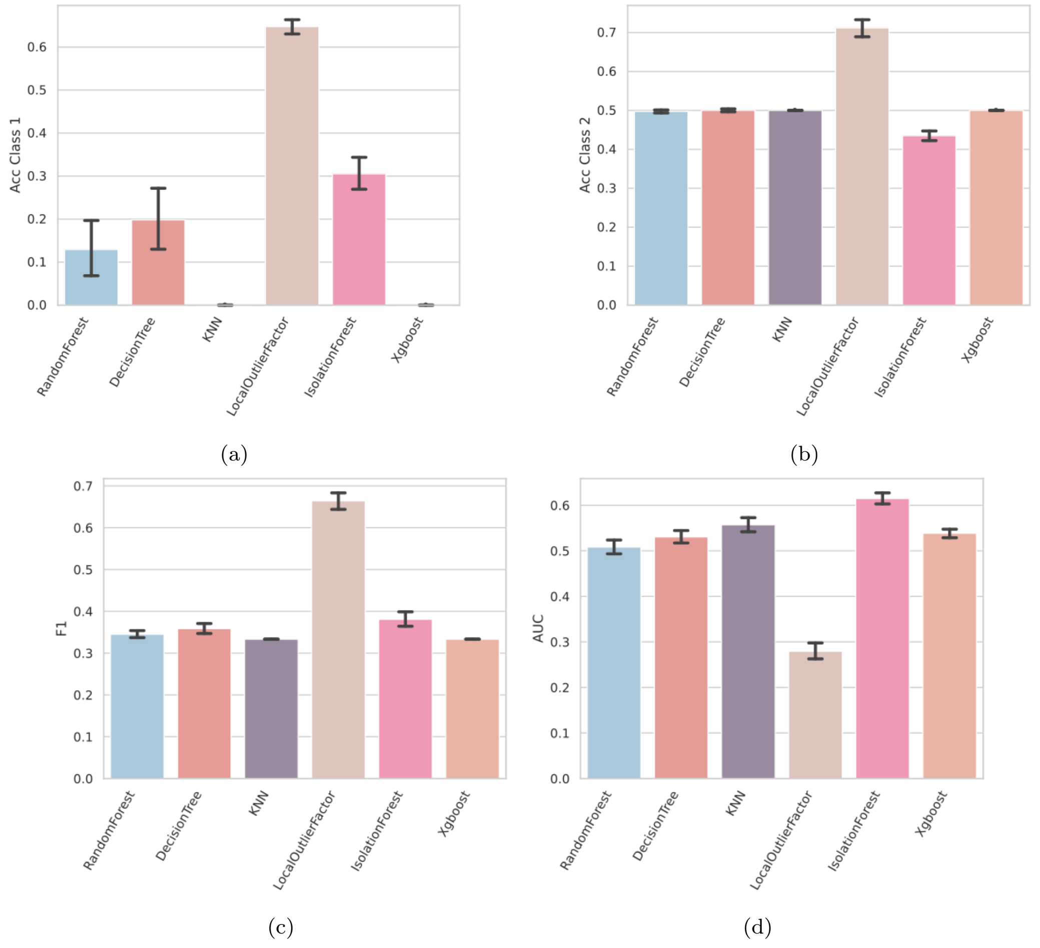

Supplementary Figure 4: Mean and Standard deviation results for all ML methods: (a) Accuracy – Class 1; (b) Accuracy – Class 2; (c) F1-measure; (d) AUC.

References

[1] N. V. Chawla, K. W. Bowyer, L. O. Hall, W. P. Kegelmeyer, Smote: synthetic minority over-sampling technique, Journal of artificial intelligence research 16 (2002) 321–357.

[2] J. Bergstra, Y. Bengio, Random search for hyper-parameter optimization., Journal of machine learning research 13 (2) (2012).

[3] T. M. Mitchell, T. M. Mitchell, Machine learning, Vol. 1, McGraw-hill New York, 1997.

[4] C. M. Bishop, N. M. Nasrabadi, Pattern recognition and machine learning, Vol. 4, Springer, 2006.

[5] L. Breiman, J. H. Friedman, R. A. Olshen, C. J. Stone, Classification and regression trees, Routledge, 2017.

[6] L. Breiman, Random forests, Machine learning 45 (1) (2001) 5–32.

[7] B. Schölkopf, A. J. Smola, F. Bach, et al., Learning with kernels: support vector machines, regularization, optimization, and beyond, MIT press, 2002.

[8] S. Haykin, Neural networks: a comprehensive foundation, Macmillan, 1994.

[9] H.-F. Yu, F.-L. Huang, C.-J. Lin, Dual coordinate descent methods for logistic regression and maximum entropy models, Machine Learning 85 (1) (2011) 41–75.

[10] D. Wunsch, R. Xu, Clustering, John Wiley & Sons, 2008.

[11] M. Ester, H.-P. Kriegel, J. Sander, X. Xu, et al., A density-based algorithm for discovering clusters in large spatial databases with noise., in: kdd, Vol. 96, 1996, pp. 226–231.

[12] F. T. Liu, K. M. Ting, Z.-H. Zhou, Isolation forest, in: 2008 eighth ieee international conference on data mining, IEEE, 2008, pp. 413–422.

[13] M. M. Breunig, H.-P. Kriegel, R. T. Ng, J. Sander, Lof: identifying density-based local outliers, in: Proceedings of the 2000 ACM SIGMOD international conference on Management of data, 2000, pp. 93–104.

[14] B. Schölkopf, J. C. Platt, J. Shawe-Taylor, A. J. Smola, R. C. Williamson, Estimating the support of a high-dimensional distribution, Neural computation 13 (7) (2001) 1443–1471.

[15] T. Fawcett, An introduction to roc analysis, Pattern recognition letters 27 (8) (2006) 861–874.
